# Sodium Disturbances in Children Admitted to a Kenyan Hospital: Magnitude, Outcome and Associated Factors

**DOI:** 10.1371/journal.pone.0161320

**Published:** 2016-09-07

**Authors:** Fredrick Ibinda, Hans-Christoph Zarnack, Charles R. Newton

**Affiliations:** 1 KEMRI-Wellcome Trust Research Programme, Centre for Geographic Medicine Research - Coast, P.O Box 230–80108, Kilifi, Kenya; 2 Department of Psychiatry, University of Oxford, Oxford, United Kingdom; Centers for Disease Control and Prevention, UNITED STATES

## Abstract

**Background:**

Perturbations of blood sodium are the most frequently encountered electrolyte disorder in sick children, and may influence fluid therapy. We examined the frequency of blood sodium perturbations, and factors and outcomes associated with hyponatremia in children admitted to a rural Kenyan hospital and investigated the risk factors associated with deaths in hyponatremic children.

**Methods:**

Plasma sodium levels and other laboratory parameters were measured in children admitted to a rural Kenyan hospital. Clinical measurements were collected using standard forms and entered into a computer database. The proportion of children admitted with hyponatremia was determined. Logistic regression models were used to investigate factors associated with hyponatremia, and death in those with hyponatremia.

**Results:**

Abnormal plasma sodium occurred in 46.6% (95% confidence interval (95%CI) 43.5–49.6%) of 1026 pediatric admissions. Hyponatremia occurred in 44.4% (95%CI 41.4–47.5%) and hypernatremia in 2.1% (95%CI 1.3–3.0%). Malaria (40.8%) was the most common underlying primary diagnosis in hyponatremic children. Malaria, hyperglycemia, wasting, high creatinine levels and preserved consciousness were associated with hyponatremia. Pallor and seizures were associated with increased mortality in hyponatremic children.

**Conclusions:**

Sodium disturbances are common in pediatric admissions to a County hospital in rural Kenya. Seizures and pallor were predictors of mortality in hyponatremic children.

## Background

Perturbations of blood concentrations of sodium are common in children admitted to hospitals and may influence fluid therapy. Recent reviews of hypotonic versus isotonic fluids highlighted the risks of hyponatremia during maintenance therapy [1, 2], which has been confirmed by a recent randomized trial in Australia [3]. However, most of these trials were conducted in Western countries, and the etiologies of dehydration and hyponatremia may be different in the resource poor countries, where the prevalence of hyponatremia may be higher.

Perturbations of blood sodium are associated with dysfunction of the central nervous system and neurological sequelae [4]. Severe hyponatremia is often associated with poor outcomes including prolonged hospital stay [5], neurological deficits and death [6, 7]. There are few studies on hyponatremia in resource limited countries, where malaria is endemic and can cause perturbation in sodium levels [8, 9]. Lack of sodium level studies in these resource limited areas may lead to improper management especially with supportive fluid therapy, which should take account electrolyte concentrations [10]. With early recognition of hyponatremia, risk factors may be ameliorated through supportive therapy to improve outcome [11]. Studies investigating the extent, associated factors and outcomes in children admitted with hyponatremia and hypernatremia are therefore warranted. There are no studies assessing the prevalence of sodium abnormalities in children admitted to district hospitals in Africa.

This study aimed at investigating the prevalence and outcomes of hyponatremia and hypernatremia in children admitted to a rural Kenyan hospital. Factors associated with hyponatremia and mortality in hyponatremic children were also investigated.

## Methods

### Study setting and population

This study was conducted in a general pediatric ward and a pediatric high dependency unit at the Kilifi County Hospital (KCH) from 1^st^ October 1999 and 15^th^ January 2000. KCH is located in the administrative centre of Kilifi County on the Kenyan Coast. Annually, KCH has about 5,000 admissions into these pediatric wards, most them (about 80%) from an area of active surveillance; the Kilifi Heath and Demographic Surveillance System (KHDSS).[12] KCH draws most of its admissions from the KHDSS residents. KCH has 35 beds in the pediatric ward and 7 beds in the pediatric high dependency unit. Severely ill children are nursed in the high dependency unit. Staff from the Kenya Medical Research Institute, which is located within the hospital, supports the KCH by providing medical services in the wards.

### Clinical definitions

Hyponatremia and hypernatremia were defined as plasma sodium <135 mmol/l and >145 mmol/l respectively. Hyponatremia was further categorically defined as mild (plasma sodium 125–135 mmol/l), moderate (115–124 mmol/l), and severe (<115 mmol/l). Table 1 provides definitions for other variables. Diagnosis of malaria was based presence of malaria parasitemia on thick and thin blood smear. The diagnosis of tetanus was clinical, based on medical history and examination, determining the presence of at least three of the following clinical findings: severe trismus, refusal to feed, generalized muscle rigidity, opisthotonus or spontaneous tetanic spasms [13, 14].

**Table 1:** Clinical definition of the variables.

Condition	Definition
Hypoglycemia	Blood glucose <2.2 mmol/l
Hyperglycemia	Blood glucose >7 mmol/l
Tachypnea	Respiratory rate >60 breaths per minute
Hypothermia	Axillary temperature <36°C
High creatinine	Creatinine >80 μmol/l
Hypokalemia	Blood potassium <3.5 mmol/l
Delayed capillary refill	>3 seconds
Low hemoglobin	Hemoglobin <8.2g/l
Hypoxia	Oxygen saturation <90% by pulse oximeter
Malnutrition	Z-score ≤2 for the anthropometric parameter weight-for-height

### Laboratory Investigations

Baseline investigations performed on site included: sodium and potassium using a Corning 614 Na+/K+ auto analyser (equipped with ion selective electrodes, CIBA-Corning Diagnostics Ltd, UK); creatinine with a Beckman creatinine analyser 2 (Beckman, Fullerton, USA); thick and thin blood films stained in 10% Giemsa for 10 minutes so that the number of asexual forms of *P*. *falciparum* could be counted per 100 white blood cells on the thick film or per 500 red blood cells on the thin film; full blood count on a M530 Coulter Counter (Luton, UK). All laboratory measurement techniques were subject to daily quality control procedures and were regularly subjected to international quality control through UKNEQAS. Systems were re-calibrated against commercially available standards if on-site measurements gave values more than 1 standard deviation away from the manufacturers mean value for the control on repeated testing.

### Management

During the period of the study, children admitted to KCH, were resuscitated with normal saline if in shock or dehydrated and then put on maintenance fluid of 4% Dextrose and 0.18% saline according to the prevailing guidelines [15]. Intravenous fluids were stopped as soon the child was able to drink and all were then allowed to eat and drink freely. If the child was still thought to be dehydrated or being mild to moderate dehydrated on admission oral rehydration solution (ORS) according to the recommendation of the World Health Organization was given. Children with severe hyponatremia were given 0.9% saline intravenously. Hypoglycemia (blood glucose < 2.2 mmol/l) was treated with 50% dextrose at 0.6 ml/kg given as a slow bolus over 5 minutes. Seizures were treated with diazepam 0.3 mg/kg given intravenously up to a maximum of three doses in 24 hours. Refractory or recurrent seizures were treated with either phenytoin (15mg/kg intravenous loading dose, 4mg/kg daily as maintenance) or phenobarbitone (15-20mg/kg intramuscular loading dose, 5mg/kg daily as maintenance). All children with fever were given either rectal or oral paracetamol (12.5mg/kg) 6 hourly as appropriate. Transfusion of 20ml/kg of whole blood was given for anemia accompanied by respiratory distress or hyperparasitemia and for severe anemia (Hb < 5g/dl).

### Ethical considerations

Informed consent was obtained from the patients’ guardians who accompanied the child to the hospital. The study was approved by Scientific and Ethics Review Unit of the Kenya Medical Research Institute.

### Statistical analysis

Data was collected using standard forms upon admission and at discharge and entered into the computer database. All analyses were performed using R; an open-source software for statistical computing and graphics (v2.15.1) [16]. Categorical variables were compared with Pearson’s Chi-square test or Fisher’s exact test when appropriate. Logistic regression models were fit to investigate associated factors for hyponatremia and death in those with hyponatremia. This was done in two steps: firstly, by fitting a univariable logistic regression to shortlist the candidate variables for the multivariable logistic model, and secondly, by including all variables with univariable p-value <0.25 into the multivariable model to establish the variables that were independently associated with the outcomes. A p-value<0.05 was considered to be statistically significant. We did not construct models for hypernatremia since it was relatively uncommon (n = 22), which would be associated with less power to measure significant associations.

## Results

### Characteristics of the study population

In the study period 1026 children were admitted to Kilifi County Hospital (KCH) of whom 540 (52.6%) were males (S1 Dataset). The median age in months (Interquartile Range) at admission was similar in males (15.4 (6.0–37.5)) and females (15.1 (6.7–39.2)). Of the admissions, 100 (9.8%) were neonates (<29 days at admission). Plasma sodium data was available for all the 1026 children. The median (Interquartile range) plasma sodium at admission was 135 (124.6–144.4) mmol/l. Three-hundred and twenty-seven (31.9%) children had a positive malaria slide (Table 2). A history of seizures was reported in 74 (7.2%) children. Twenty-four (5.5%) of the children had seizures while in the hospital. Of the 1026 admissions, 81 (7.9%) died in the hospital.

**Table 2:** Characteristics of study participants.

Characteristics	Hyponatremic, n (%)	Hypernatremic, n (%)	Normonatraemic, n (%)	All admissions, n (%)
All	456 (44.4%)	22 (2.1%)	548 (53.4%)	1026 (100%)
Malnutrition (WHZ<−2)*	95 (23.4)	2 (25.0)	96 (19.79)	193 (21.47)
Hypoxia	39 (8.67)	6 (27.27)	39 (7.21)	84 (8.29)
Hypothermia	18 (3.96)	1 (4.55)	28 (5.11)	47 (4.59)
Unconscious	26 (5.70)	1 (4.55)	42 (7.66)	69 (6.73)
Deep breathing	26 (5.70)	2 (9.09)	36 (6.57)	64 (6.24)
Chest indrawing	90 (19.74)	2 (9.09)	121 (22.08)	213 (20.76)
Stiff neck	15 (3.30)	6 (27.27)	20 (3.66)	41(4.01)
Vomiting	137 (30.04)	5 (22.73)	135 (24.64)	277 (27.00)
Delayed capillary refill	73 (16.82)	5 (22.73)	86 (16.44)	164 (16.75)
Cyanosis	9 (1.97)	-	7 (1.28)	16 (1.56)
Seizures in hospital	24 (5.54)	1 (4.76)	37 (7.14)	62 (6.38)
Pallor	57 (12.50)	3 (13.64)	64 (11.68)	124 (12.09)
Skin turgor	29 (6.36)	3 (13.64)	18 (3.28)	50 (4.87)
Skin/hair malnutrition	25 (5.48)	1 (4.55)	25 (4.56)	51 (4.97)
Hypoglycemia	297 (65.13)	6 (27.27)	359 (65.51)	662 (64.52)
Hyperglycemia	41 (8.99)	1 (4.55)	34 (6.20)	76 (7.41)
Hypokalemia	71 (15.57)	5 (22.73)	74 (13.50)	150 (14.62)
Malaria	186 (40.79)	3 (13.64)	138 (25.18)	327 (31.87)
High creatinine	31 (6.80)	13 (59.09)	19 (3.47)	63 (6.14)
Acidosis	41 (8.99)	8 (36.36)	53 (9.67)	102 (9.94)
Wasting	31 (6.80)	1 (4.55)	17 (3.10)	49 (4.78)
Diarrhoea	97 (21.27)	3 (13.64)	89 (16.24)	189 (18.42)
Low hemoglobin	252 (55.26)	5 (22.73)	217 (39.6)	474 (46.20)

*weight for height z-score

### Proportion of sodium disturbances

Out of the 1026 admissions, 478 (46.6% (95% confidence interval (95%CI) 43.5–49.6%)) children had blood sodium disturbances with 456 (44.4% (95%CI 41.4–47.5%)) hyponatremic and 22 (2.1% (95%CI 1.3–3.0%)) hypernatremic. Most children had mild (41.9%, 95%CI 38.9–44.9%) rather than moderate (2.2%, 95%CI 1.3–3.1%) or severe hyponatremia (0.3%, 95%CI 0.06–0.8%). Neonates were found to be have increased risk of hypernatremia compared to non-neonates (68.2% vs 38.1%, p<0.001), but not hyponatremia (11.2% vs 7.9%, p = 0.075). There was an evidence of increased odds of hyponatremia in females than males (Odds ratio (OR) 1.29, 95%CI 1.01–1.65, p = 0.044) but not for hypernatremia (OR 1.34, 95%CI 0.57–3.21, p = 0.497).

### Primary diagnosis for hyponatremic and hypernatremic children

**Fig 1:**
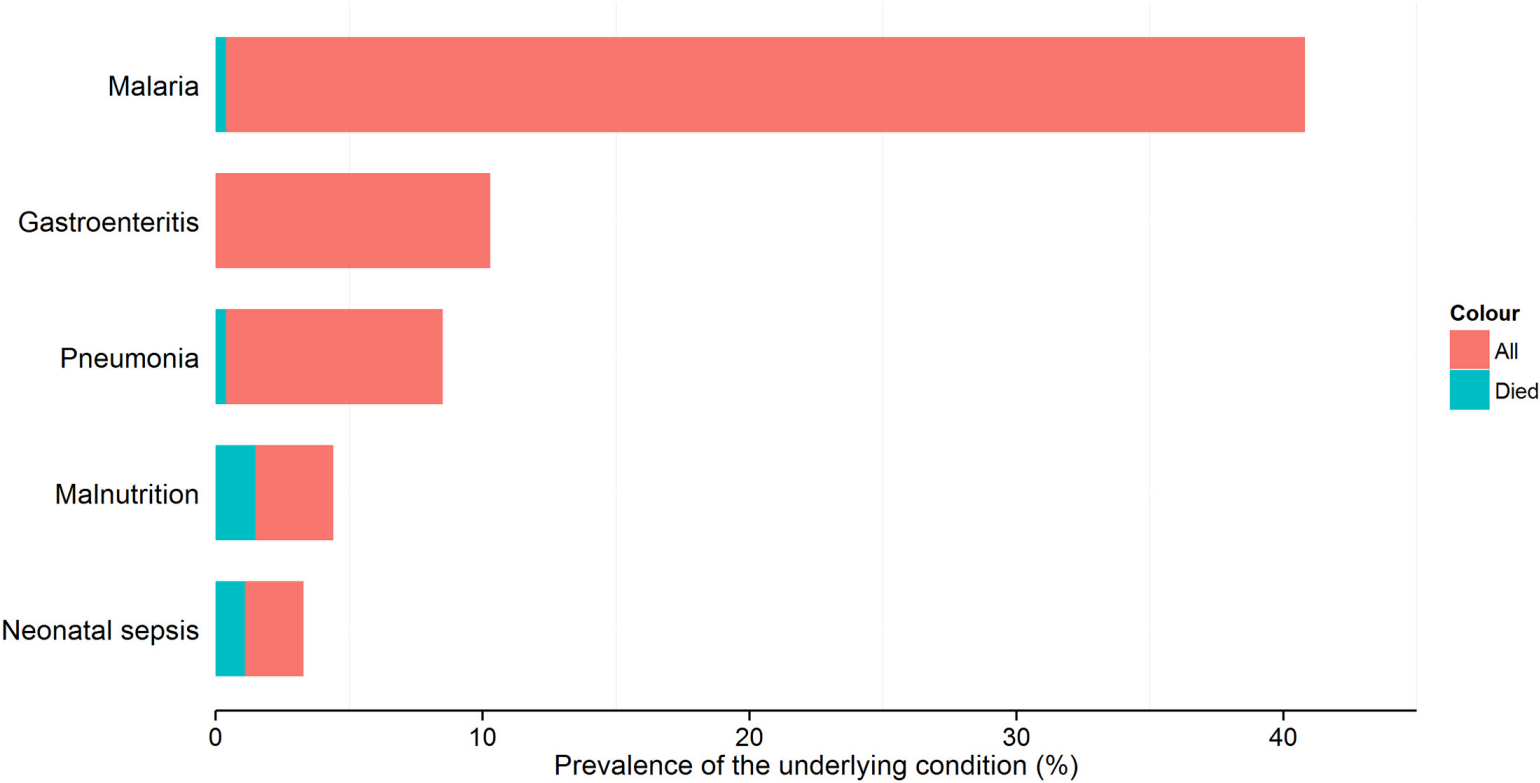
Most common underlying conditions in children with hyponatremia. This figure shows the most common underlying conditions in hyponatremic children. Red shows the percentage of children with the condition while blue represents the percentage that died from that condition.

**Fig 2:**
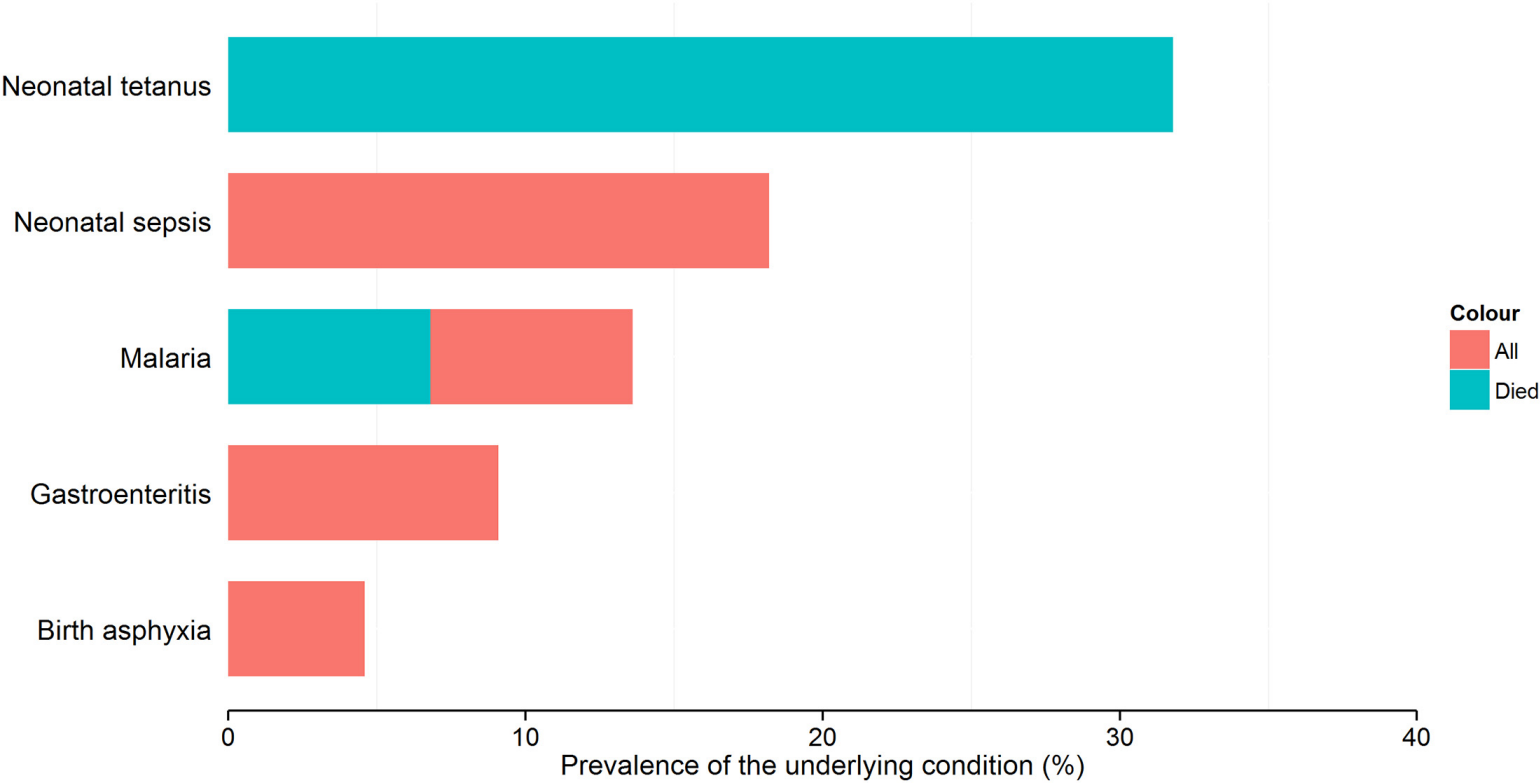
Most common underlying conditions in children with hypernatremia. This figure shows the most common underlying conditions in hypernatremic children. Red shows the percentage of children with the condition while blue represents the percentage that died from that condition. A completely blue bar means that all children with that condition died.

The most common underlying conditions in hyponatremic children were: malaria (n = 186; 40.8%), gastroenteritis (n = 47; 10.3%), and pneumonia (n = 39; 8.6%) (Fig 1). In hypernatremic children, the most prevalent underlying conditions were: neonatal tetanus (n = 7; 31.8%), neonatal sepsis (n = 4; 18.2%) and malaria (n = 3; 13.6%) (Fig 2). Hyponatremia was significantly more frequent in those with malaria compared to those without (Chi-square test; p<0.001). Eight (36.4%) children with hypernatremia died including all the children who had neonatal tetanus as the underlying disease (Fig 2). We had data about seizures in six of the eight children who died and all of them had seizures while in the hospital. Malaria was associated with seizures in both normonatremic (16.3% vs 4.0%) and hyponatremic (6.7% vs 4.7%) children.

### Factors associated with hyponatremia

Several variables were studied for possible association with hyponatremia in the univariable analysis (Table 3). Only nine of the ten variables with a p-value<0.25 were included in the multivariable logistic regression model in order to identify the ones that were independently associated with hyponatremia. Malnutrition was not included in the multivariable model since it was highly correlated with wasting (p<0.001). From the multivariable model, malaria (OR 2.25 (95%CI 1.67–3.03), p<0.001), high creatinine (OR 2.65 (95%CI 1.41–5.09), p = 0.003), wasting (OR 2.43 (95%CI 1.29–4.70), p = 0.007), impaired consciousness (OR 0.41 (95%CI 0.23–0.71), p = 0.002), hyperglycemia (OR 1.72 (95%CI 1.03–2.87), p = 0.038) and low hemoglobin levels (OR 1.68 (95%CI 1.28–2.21), p<0.001) were independently associated with hyponatremia.

**Table 3:** Factors associated with hyponatremia.

Variables	Univariable analysis		Multivariable analysis	
	Odds Ratio (95%CI)	p value	Odds Ratio (95%CI)	p value
Malnutrition (WHZ<−2)*	1.24 (0.90–1.71)	0.192	-	-
Hypoxia	1.22 (0.77–1.94)	0.397	-	-
Hypothermia	0.76 (0.41–1.39)	0.386	-	-
Unconscious	0.73 (0.43–1.20)	**0.219**	0.41 (0.23–0.71)	**0.002**
Deep breathing	0.86 (0.51–1.44)	0.570	-	-
Chest indrawing	0.87(0.64–1.18)	0.364	-	-
Stiff neck	0.90 (0.45–1.76)	0.754	-	-
Vomiting	1.31 (0.99–1.74)	**0.055**	1.17 (0.85–1.61)	0.343
Delayed capillary refill	1.03 (0.73–1.45)	0.876	-	-
Cyanosis	1.56 (0.58–4.39)	0.384	-	-
Seizures in hospital	0.76 (0.44–1.29)	0.317	-	-
Pallor	1.08 (0.74–1.58)	0.691	-	-
Skin turgor	2.00 (1.11–3.71)	**0.024**	1.66 (0.85–3.30)	0.144
Skin/hair malnutrition	1.21 (0.68–2.15)	0.505	-	-
Hypoglycemia	0.98 (0.76–1.28)	0.900	-	-
Hyperglycemia	1.49 (0.93–2.41)	**0.096**	1.72 (1.03–2.87)	**0.038**
Hypokalemia	1.18 (0.83–1.68)	0.354	-	-
Malaria	2.05 (1.57–2.68)	**<0.001**	2.25 (1.67–3.03)	**<0.001**
High creatinine	2.03 (1.14–3.71)	**0.018**	2.65 (1.41–5.09)	**0.003**
Acidosis	0.92 (0.60–1.41)	0.713	-	-
Wasting	2.28 (1.26–4.26)	**0.008**	2.43 (1.29–4.70)	**0.007**
Diarrhoea	1.39 (1.01–1.92)	**0.042**	1.34 (0.92–1.91)	0.131
Low hemoglobin	1.88 (1.47–2.43)	**<0.001**	1.68 (1.28–2.21)	**<0.001**

CI, confidence interval;

*weight for height z-score;

### Factors associated with death in hyponatremia

There were 39 (8.6%) deaths among the children with hyponatremia compared to 34 (6.2%) deaths were recorded in normonatremic children. Mortality in hyponatremic children was not statistically different from that in normonatremic children (p = 0.192). Several variables were investigated for an association with mortality in hyponatremic children in the univariable analysis (Table 4). Eighteen of these variables had a p-value<0.25 and seventeen of them were included in the multivariable logistic regression in order to identify the variables that were independently associated with death in hyponatremia. Malnutrition was not included in the multivariable model since it was highly correlated with wasting (p<0.001). From the multivariable model, pallor (OR 4.17 (95%CI 1.12–14.17), p = 0.025), and seizures while in the hospital (OR 7.04 (95%CI 1.43–31.23), p = 0.011) were found to be associated with increased mortality in hyponatremia. However, hyponatremic children who had malaria had reduced mortality compared to those without malaria (OR 0.10 (95%CI 0.01–0.45), p = 0.010).

**Table 4:** Factors associated with death in hyponatremia.

Variables	Univariable analysis				Multivariable analysis	
	Died, n (%)	Survived, n (%)	Odds ratio (95%CI)	p value	Odds ratio (95%CI)	p value
Malnutrition (WHZ<−2)*	9 (42.86)	86 (22.34)	2.61 (1.03–6.37)	**0.036**	**-**	**-**
Hypoxia	10 (27.78)	29 (7.00)	5.11 (2.17–11.38)	**<0.001**	1.31 (0.28–4.92)	0.706
Hypothermia	6 (15.79)	12 (2.88)	6.33 (2.09–17.47)	**0.001**	2.60 (0.51–11.55)	0.220
Unconscious	7 (17.95)	19 (4.56)	4.58 (1.69–11.31)	**0.001**	1.12 (0.12–9.02)	0.914
Deep breathing	5 (12.82)	21 (5.04)	2.77 (0.88–7.32)	**0.054**	0.55 (0.08–2.60)	0.492
Chest indrawing	16 (41.03)	74 (17.75)	3.22 (1.60–6.37)	**0.001**	1.98 (0.70–5.45)	0.187
Stiff neck	2 (5.26)	13 (3.12)	1.73 (0.26–6.58)	0.483	-	-
Vomiting	9 (23.08)	128 (30.70)	0.68 (0.30–1.41)	0.324	-	-
Delayed capillary refill	13 (38.24)	60 (15.00)	3.51 (1.63–7.31)	**0.001**	1.75 (0.58–5.01)	0.308
Cyanosis	4 (10.26)	5 (1.20)	9.42 (2.24–37.17)	**0.001**	3.10 (0.32–26.96)	0.310
Seizures in hospital	6 (17.14)	18 (4.52)	4.37 (1.49–11.35)	**0.004**	7.47 (1.47–34.59)	**0.011**
Pallor	9 (23.08)	48 (11.51)	2.31 (0.98–4.98)	**0.041**	3.93 (1.07–13.27)	**0.031**
Skin turgor	10 (25.64)	19 (4.56)	7.22 (2.99–16.74)	**<0.001**	1.97 (0.39–8.65)	0.389
Skin/hair malnutrition	6 (15.38)	19 (4.56)	3.81 (1.31–9.72)	**0.008**	0.94 (0.13–5.57)	0.946
Hypoglycemia	18 (46.15)	279 (66.91)	0.42 (0.22–0.82)	**0.011**	0.62 (0.19–2.05)	0.414
Hyperglycemia	9 (23.08)	32 (7.67)	3.61 (1.51–8.02)	**0.002**	1.80 (0.34–8.64)	0.470
Hypokalemia	7 (17.95)	64 (15.35)	1.21 (0.47–2.71)	0.669	-	-
Malaria	2 (5.13)	184 (44.12)	0.07 (0.01–0.23)	**<0.001**	0.10 (0.01–0.47)	**0.010**
High creatinine	12 (30.77)	19 (4.56)	9.31 (4.03–21.07)	**<0.001**	3.21 (0.83–11.58)	0.079
Acidosis	11 (28.21)	30 (7.19)	5.07(2.23–10.98)	**<0.001**	1.56 (0.40–5.36)	0.501
Wasting	9 (23.08)	22 (5.28)	5.39(2.19–12.45)	**<0.001**	2.29 (0.47–9.71)	0.277
Diarrhoea	6 (15.38)	91 (21.82)	0.65 (0.24–1.50)	0.351	-	-
Low hemoglobin	21 (53.85)	231 (55.40)	0.94 (0.49–1.83)	0.852	-	-

CI, confidence interval;

*weight for height z-score (whz), this variable was not included in the multivariable model since it was highly correlated with wasting.

## Discussion

We found that slightly under half (47%) of the admissions had sodium perturbations, predominantly hyponatremia (44%). Malaria was the most common underlying primary diagnosis in hyponatremic. Malaria, wasting, hyperglycemia, low hemoglobin and high creatinine levels were associated with hyponatremia. Pallor and seizures were associated with increased mortality in children with hyponatremia.

This study confirms that sodium disturbances are common in pediatric admissions, particularly hyponatremia. Although the proportion of children admitted with severe hyponatremia was low, it is of clinical importance because it has been reported to be associated with poor outcomes including mortality [17].

### Hyponatremia and underlying conditions

Hyponatremia is common in adult and pediatric patients with severe malaria [18]. Previous studies performed on the Kenyan Coast have shown that over 50% of children admitted with severe malaria had hyponatremia, which is similar with this study [8, 9]. The results from that previous study [8] showed that hyponatremic children are less water depleted, often showing appropriate rather than inappropriate secretion of antidiuretic hormone. Hyponatremia did not impair the conscious level in all children in this study, as documented in a study of children with severe malaria [19]. Cerebral salt wasting was documented in some children with cerebral malaria [8, 9], but not in this study.

### Hypernatremia and underlying conditions and associated factors

We found that hypernatremia was common in neonatal tetanus, and that all these neonates who had this condition died. Neonatal tetanus can possibly be attributed to traditional delivery practices and can be easily prevented according to recent study from this area [14].

The association between high creatinine levels and hyponatremia suggest that dehydration with pre-renal impairment is common. Wasting is a measure of malnutrition, which compromises the capacity to handle water overload particularly in infants [20] This analysis confirms the well-established association between hyperglycemia and hyponatremia [21], driven by the high prevalence of malnutrition and dehydration in this study.

### Fatality in children with hyponatremia and hypernatremia

The overall mortality rate in hypernatremic children was higher than in hyponatremic children and was similar with normonatremic children. The observed fatality rate in hypernatremic children is higher than that observed in Texas [22] and elsewhere [23, 24]. This could possibly be ascribed to the high neonatal hypernatremia, which is secondary to highly preventable conditions like neonatal tetanus as observed in this study or other many important causes of mortality in African children [25].

### Factors associated with death in children with hyponatremia

In this study, pallor was associated with increased mortality. Although pallor is most commonly associated with anemia, also it is caused by hypoglycemia and shock, which are commonly seen in very sick children, associated with death.

Seizures were identified as an important predictor for mortality in hyponatremic children compared to those with normal sodium levels. Prepubescent children have been shown to have poor outcome associated with hyponatremic encephalopathy [26]. Seizures are a manifestation of hyponatremic encephalopathy, although some may have been due to malaria [27]. In Sweden, seizures have been documented as the only neurologic manifestation of hyponatremia in patients with low serum sodium levels who did not have a prior diagnosis of epilepsy [28]. In malaria endemic areas, malaria is a major cause of seizures [27], which are usually complicated and associated with status epilepticus [29], often leading to neurological impairments [30] and development of epilepsy [31]. Status epilepticus has been documented as one of the parameters for recognising hyponatremic seizures [32] In a recent study, most complications such as convulsive status epilepticus were found to be more common in children with seizures and can be fatal [33].

It is not clear why malaria was associated with reduced mortality in hyponatremia in this study. Similar finding were found in a study conducted on adults with severe malaria [19]. In our study and that of Hanson and colleagues [19], consciousness was more preserved in hyponatremic compared to normonatremic patients. This could be one the reason why mortality was reduced in hyponatremic patients with malaria—impaired consciousness has been documented as a strong predictor for mortality in malaria [34, 35].

Put together, seizures increased the probability of mortality whilst malaria reduced mortality in hyponatremic children. We hypothesize that mortality in those with seizures was increased due to complications arising from convulsive status epilepticus. In a previous study conducted in Kilifi, the authors found that mortality in children with convulsive status epilepticus was lower in those with malaria compared to those without [29]. The authors attributed their finding to a possibility of a more serious underlying condition such as encephalitis, which was associated with worse outcome in their study [29]. It is therefore possible that children with malaria are more easily treated or hyponatremic children without malaria have more serious underlying conditions. A study has shown that hyponatremia in children with severe malaria is unrelated to severe problems like abnormal renal function [9]. Studies are needed to validate these hypotheses and guide future interventions.

This study has the following limitations. Firstly, there are multiple possible etiologies of hyponatremia. Distinguishing among these possibilities requires measurement of urine sodium and urine osmolality, which was not done and therefore the etiology of the hyponatremia is uncertain. Secondly, no follow-up data (apart from the discharge status) on these children is provided hence it is not known how many children developed complications or neurological sequelae attributable to hyponatremia and hypernatremia.

### Conclusion

Perturbation of blood sodium is common in rural Kenyan pediatric admissions. Hyponatremia is associated with malaria, wasting, hyperglycemia, low hemoglobin and high creatinine levels. Neonates are at increased risk of hypernatremia, particularly those with neonatal tetanus. Seizures and pallor are predictors of poor prognosis in hyponatremic children.

## Supporting Information

S1 Dataset Dataset of subject’s information. (DTA)
